# Improved optogenetic modification of spiral ganglion neurons for future optical cochlear implants

**DOI:** 10.7150/thno.104474

**Published:** 2025-03-18

**Authors:** Anupriya Thirumalai, Jana Henseler, Marzieh Enayati, Kathrin Kusch, Roland Hessler, Tobias Moser, Antoine Tarquin Huet

**Affiliations:** 1 Institute for Auditory Neuroscience, University Medical Center Göttingen, 37075 Göttingen, Germany.; 2 InnerEarLab, University Medical Center Göttingen, 37075 Göttingen, Germany.; 3 Auditory Circuit Lab, University Medical Center Göttingen, 37075 Göttingen, Germany.; 4 Göttingen Graduate School for Neurosciences and Molecular Biosciences, University of Göttingen, Göttingen, Germany.; 5 Auditory Neuroscience and Optogenetics Laboratory, German Primate Center, Göttingen, Germany.; 6 Functional Auditory Genomics group, University Medical Center Göttingen, 37075 Göttingen, Germany.; 7 Else Kroener Fresenius Center for Optogenetic Therapies, University Medical Center Göttingen, 37075 Göttingen, Germany.; 8 Cluster of Excellence "Multiscale Bioimaging: from Molecular Machines to Networks of Excitable Cells" (MBExC), University of Göttingen, Göttingen, Germany.; 9 Auditory Neuroscience and Synaptic Nanophysiology Group, Max Planck Institute for Multidisciplinary Sciences, Göttingen, Germany.; 10 Current address: Institute for Neurosciences Montpellier, Institut National de la Santé et de la Recherche Médical, University of Montpellier, Montpellier, France.

**Keywords:** optical cochlear implants, viral vector delivery, microcatheter-based administration, cochlear optogenetic stimulation, spiral ganglion neurons.

## Abstract

Optogenetic stimulation has become a promising approach for restoring lost body function. For example, partial restoration of vision has been achieved in a blind patient and preclinical proof-of-concept has been demonstrated for optogenetic hearing restoration. In preparation for clinical translation of hearing restoration, efficient and safe optogenetic modification of spiral ganglion neurons (SGNs) in the mature cochlea remained to be developed.

**Methods:** Here, we established microcatheter-based administration of adeno-associated virus (AAV) into *scala tympani* of the cochlea of Mongolian gerbils and compared it to the previously developed direct AAV-injection into the spiral ganglion. We probed the potential of AAV-PHP.S to express channelrhodopsins (ChRs) under the control of the human synapsin promotor in mature SGNs of hearing and deafened gerbils.

**Results:** Using the microcatheter approach, but not with the AAV-modiolus injection, we achieved reliable ChR expression in SGN enabling optogenetic stimulation of the auditory pathway in 80% of the treated animals. Yet, the efficiency of SGN transduction was modest with only ~30% ChR-expressing SGNs. Moreover, we encountered off-target expression in hair cells in hearing gerbils in both approaches. We did not detect ChR expression in the central nervous system using microcatheter administration. Comparing optogenetic auditory brainstem responses of gerbils with and without hair cell transduction confirmed that SGNs were the primary site of optogenetic stimulation of the pathway.

**Conclusions:** The AAV.PHP-S microcatheter administration via the round window with pressure relief at the round window is a reliable approach to optogenetically modify the SGNs in order to restore hearing with future optical cochlear implants.

## Introduction

More than 5% of the world's population suffers from disabling hearing impairments, and the majority of them are affected from what is known as sensorineural hearing loss 1. In these patients, the hair cells that transduce the sound into micromechanical amplification (outer hair cells) or release of glutamate (inner hair cells, IHC) that activates the spiral ganglion neurons (SGN) are dysfunctional or absent. Despite recent clinical advances in cell therapy for otoferlin-related deafness 2,3, hearing aids and cochlear implants will remain the pillar of hearing restoration for the foreseeable future because of their one-size-fits-all nature. Hearing restoration for severely hearing-impaired patients relies on the use of electrical cochlear implants (eCI), that bypass the lost or dysfunctional cochlear hair cells and directly stimulate the SGNs that are typically maintained for longer. By restoring hearing perception to the vast majority of its currently more than a million users, the eCI is considered to be the most successful neuroprosthesis to date (for review, see 4). While eCI enables the perception of speech in a quiet environment, users recognize an unmet medical need in the real-life condition where background noise competes with speech 5,6. This deficiency is commonly attributed to the wide current spread from each electrode contact in the saline-filled cochlea, activating large SGN groups and limiting spectral information transfer 7. Despite engineering efforts to reduce the spread of electrical stimulation, clinical benefits have remained limited. An alternative way to achieve a spatially narrower activation of the SGNs, and thus to increase the spectral information transfer, is to switch the stimulation modality to light, since light can be better spatially confined 8-10. In this approach, photocontrol is achieved by driving viral vector-mediated expression of channelrhodopsin (ChR) in the SGNs. This concept leads to the current development of an optical cochlear implant (oCI) consisting of a medical device on the one hand and an optogene therapy on the other (for review, see 11).

For the oCI to be translated into a clinical approach, efficiency, reliability and safety of optogene therapy needs to be demonstrated in preclinical work. Previous work has shown that efficient optogene therapy can be achieved by injection of a viral vector carrying ChR under the control of the human synapsin promoter (hSyn) into *scala tympani* of early postnatal rodents (i.e. “intrascalar”, 12-16). At this age of optogene therapy, a broad range of viral capsids was evaluated and reached high rates of ChR transduction in the SGNs (≥~70%: AAV2/6 12,14,17,18, AAV2/9 19, AAV PHP.B 10,13,14,20,21, AAV PHP.eB 20 and AAV.Anc80 15,16). A single AAV administration was shown by several rodent studies to result in long-lasting ChR transduction in SGNs 12,17,18. For example, early postnatal optogene therapy in mice was demonstrated to be stable for two years (the lifetime of mice) but associated with extracochlear ChR expression at different levels of the nervous system 17. ChR expression in SGN adult cochleae can also be achieved by direct injection of the viral vector into the cochlear modiolus (“intramodiolar”, directly into the Rosenthal canal of the bony modiolus that houses the spiral ganglion, 18). The success of the optogene therapy for enabling optogenetic SGN stimulation at this age was limited to ~40% of the treated gerbils and a low ChR expression rate (~20-30% SGNs) in the responsive cochleae 8,9,14,18,22. In addition, intramodiolar pressure injection caused a loss of ~20% of the SGNs 18. Even less efficient and reliable transduction was reported upon intrascalar AAV injection in adult animals 15.

In this study, we aimed to develop a translatable delivery approach and to identify appropriate viral vectors that would allow ChR expression in the SGNs of all treated adult animals as relevant for future clinical work. Previous work in adult mice demonstrated the role of the cochlear aqueduct in the spread of drugs locally administered in the cochlea towards the brain 23. For this purpose, we selected the capsid AAV.PHP-S for its tropism towards the peripheral nervous system 24, thus aiming to avoid the transduction of neurons in the central nervous system in the event that the viral suspension would spread towards the brain. First, we evaluated the capsid AAV.PHP-S for its ability to mediate ChR expression in young and adult SGNs employing the Mongolian gerbil for its large cochlea 25. We then tested multiple administration approaches that allow for the replacement of cochlear fluids in a standardized and controllable manner and compared them to the currently established optogene therapy for adult animals via intramodiolar injection. We found that slow delivery of AAV.PHP-S via a microcatheter inserted into the round window in combination with an evacuation vent at the oval window (RW_µ-cat_ + vent) allowed expression of ChR in all treated cochleae and that optically evoked auditory potentials could be recorded in ≥80% of the treated cochleae. Finally, in a pharmacological model of profound sensorineural hearing loss, we demonstrated that successful optogenetic stimulation of the auditory pathway following AAV-mediated transduction of SGNs by RW_µ-cat_ + vent administration does not require the presence of IHCs.

## Results

### AAV.PHP-S as a viral vector for optogenetic modification of spiral ganglion neurons

**Figure 1:**
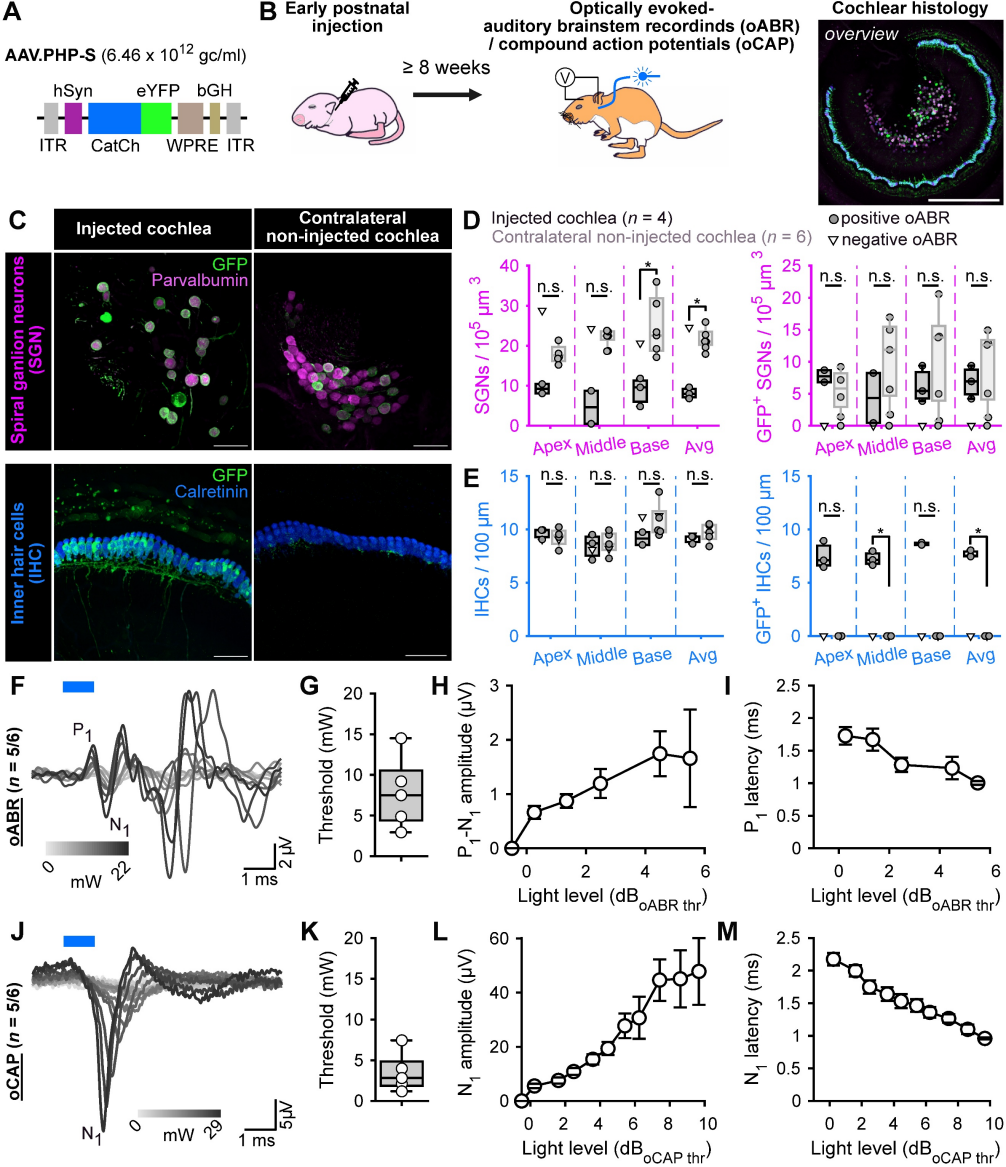
AAV.PHP-S mediates efficient ChR-expression of CatCh in SGNs following cochlear injection at early postnatal age. **A.** pAAV plasmid used in this study containing CatCh fused with eYFP. Expression was driven by human synapsin promoter (hSyn) and enhanced by the Woodchuck hepatitis virus post-translational regulatory element (WPRE) and bovine growth hormone (bGH). ITR corresponds to the inverted terminal repeats. **B.** Time course of the experiment: At early postnatal age, animals received an intracochlear viral suspension injection. At least 8 weeks later, optically evoked auditory brainstem responses (oABR) were measured and the cochleae were collected for histology. **C.** Representative maximum projection of confocal images obtained from immunolabelled cross-modiolar sections of an injected (left) and a non-injected cochlea (right, scale bar = 50 µm). GFP (green) marks ChR-expressing cells. The first row corresponds to the spiral ganglion neurons (SGN) labelled with parvalbumin (magenta). The second row corresponds to the inner hair cells (IHC) labelled with calretinin (blue). **D-E.** Quantification of the SGN density, GFP^+^ SGN density (D), IHC density and GFP^+^ IHC density (E). Data from the injected cochleae are represented in black, and contralateral non-injected cochlea in grey. For the injected cochlea, a grey-filled marker was used when positive oABRs were measured and an open-marker for the negative oABRs. Wilcoxon rank sum test (n.s., non-significant; *, *P* ≤ 0.05). **F.J.** Representative oABRs (F) and optically evoked compound action potentials (oCAP). The first wave of both potentials reflects the synchronous activation of the SGNs. **G-I, K-M.** Quantification of the activation thresholds (G,K), first wave amplitude (H,L) and first wave latency (I,M) of the oABR (G-I, *n* = 5 cochleae) and the oCAP (K-M, *n* = 5 cochleae). The potential latencies and amplitudes are expressed as mean ± SEM as a function of the light level above threshold (see Material and methods for details). Box plots show minimum, 25^th^ percentile, median, 75^th^ percentile, and maximum.

Here, we evaluated AAV.PHP-S, a capsid designed to specifically transduce neurons from the peripheral nervous system (Chan *et al.*, 2017), for its ability to mediate ChR expression in the SGNs. AAV.PHP-S was first evaluated following pressure injection into the cochlea of early postnatal age gerbils (7-8 postnatal day), which, at this age, is associated with high SGN transduction rate in most of injected gerbils 14. We expressed the blue-light activated ChR2-variant, CatCh 18,26, fused to the enhanced yellow fluorescent protein (eYFP) under control of hSyn promoter (**Figure 1A**). The capsid was injected at a titer of 6.46 x 10^12^ genome copies per milliliter (gc/mL). Approximately, 8 weeks after injection, success of the optogene therapy was assessed. The histological expression of CatCh-eYFP was analysed by confocal microscopy of cross-modiolar section immunolabeled for GFP, using parvalbumin as a SGN marker and calretinin as an inner hair cell (IHC) marker for, both, the injected and non-injected cochlea. To observe SGNs and the IHCs they innervate, we developed the semi-thick cross-modiolar section of the cochlea (thickness = 220 µm, **Figure 1B-C**). For efficient analysis of the large number of cells in the images, the SGNs were automatically segmented in 3 dimensions using a self-trained model in CellPose 2.0 27 of Arivis 4D software (see the section “Material and methods” for details). ChR-expressing SGNs (GFP^+^ SGNs) were semi-automatically identified based on the distribution of averaged GFP signal per 3D segmented cell 14,21.

The optical activation of the peripheral auditory pathway was validated by measuring optically evoked auditory brainstem responses (oABRs, **Figure 1F**). In 5 out of the 6 injected cochleae, oABRs could be elicited and, in 3 of the 4 injected cochleae analysed histologically, ChR-expressing SGNs were found. In the oABR negative cochlea, no ChR-expressing SGNs were found and thus, we hypothesized that this cochlea must have been mis-injected and its data will only be displayed but not included in the following quantification. The SGN density of the injected cochleae was significantly reduced compared to the contralateral non-injected cochleae (**Figure 1C-D**, injected: 8.12 ± 0.81, *n* = 3 and non-injected 21.73 ± 1.31 SGNs / 10^5^ µm^3^, *n* = 5, *P* = 0.036, Wilcoxon rank). Our previous study reported a slight decrease of SGNs, potentially induced by a transient increase of pressure into the cochlea during the injection 14, but the reduction was stronger in the current study.

The density of GFP^+^ SGNs was similar between the cochleae and amounted to 6.83 ± 1.49 and 9.35 ± 2.63 SGNs / 10^5^ µm^3^ for injected and non-injected cochlea, respectively (**Figure 1C,D**). To take into account the difference of SGNs density between both sides, ChR-expression was also quantified as the ratio between the number of GFP^+^ SGNs and the total number of residual SGNs (i.e., the ChR-expression rate). In this case, the transduction rate tended to be higher for the injected cochleae (82.17% ± 10.54) compared to the contralateral non-injected ones (45.3% ± 13.08, *P* = 0.14, Wilcoxon rank sum test).

Contrary to previous reports using other AAV-capsids (AAV2/6: 18; AAV.PHP.B: 14), AAV.PHP-S administration was associated with IHCs that also expressed CatCh-eYFP and thus we quantified the extent of their ChR-expression. The IHC density was similar between both ears and amounted to 9.18 ± 0.18 and 9.64 ± 0.21 IHC / 100 µm for the injected (*n* = 5) and the contralateral non-injected (*n* = 6) cochleae. Nonetheless, ChR-expressing IHCs were solely limited to the injected cochlea (7.67 ± 0.15 GFP^+^ IHC / 100 µm, i.e. ChR-expression rate = 85.47 ± 1.47%). We assume that the cross-modiolar slicing facilitates the detection of ChR-expressing IHCs, compared to the mid-modiolar sections used in the previous studies.

Next, we recorded oABRs from scalp electrodes in response to 1 ms light pulses delivered into the cochlea, at 10 Hz, by an optical fiber (⌀ = 200 µm) coupled to a 488 nm laser (**Figure 1B**). At low light intensities, the oABRs were characterized by 2-3 positive waves as previously described in gerbils 8,9,14,18. At higher light intensities, the oABRs were contaminated by a large and late likely non-auditory potential, potentially reflecting facial nerve activation (**Figure 1F**). The oABR threshold on average amounted to 7.8 ± 1.99 mW (**Figure 1G**). To take into account the large distribution of oABR thresholds, quantification of the oABR wave amplitudes and latencies was related to light levels relative to the oABR threshold using the following formula:



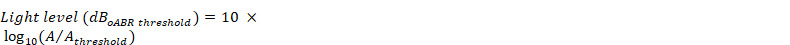



where A is the radiant flux and A_threshold_ the radiant flux at the oABR threshold in mW. The amplitude of the first oABR wave increased linearly with the light level (**Figure 1H**) while the first wave latency decreased (**Figure 1I**) up to ~4 dB of radiant flux. To characterize SGN responses to a wider range of light levels regardless of the absence or presence of non-auditory potentials, optically evoked compound action potentials (oCAP) were recorded using an electrode placed at the surface of the cochlea (**Figure 1J**). The oCAPs reflect the synchronous activation of the SGNs and are recorded from a silver ball electrode implanted in the RW niche 28,29. The oCAP thresholds tended to be lower than those of oABRs measured from the same animal and amounted to 3.5 ± 1.09 mW (**Figure 1K**, *P* = 0.06, *n* = 5, Wilcoxon signed rank test). The extent of cochlear activation, reflected by the oCAP wave amplitude, increased with the radiant flux for up to 7 dB and seemed to saturate above (**Figure 1L**). Nonetheless, the oCAP latency decreased over the whole tested range (> 9 dB, **Figure 1M**).

SGN loss and transduction of IHCs in the injected cochlea might indicate that the administered dose of AAV-PHP.S-hSyn-CatCh-eYFP was too high for achieving safe and specific optogene therapy. Alternatively, AAV-PHP.S might differ from that of previously tested AAVs in tropism or show cell-type specific toxicity.

### Development of the RW_µ-cat_ + OW approach to improve the reliability of optogene therapy in adults

While AAV injection into the *scala tympani* at early postnatal age results in reliable transduction and ChR expression of SGNs, adult intramodiolar injection is characterized by a poorer outcome, with functionally relevant SGN transduction being achieved in only a fraction of the treated cochlea 8,14,18,22. Moreover, while surgically feasible in humans 30, a round window approach might represent the first choice, given its frequent use in CI surgery, its successful use for clinical cochlear gene therapy targeting IHCs 3,31 and the availability of clinically-approved microcatheters (µ_cat_). However, to our knowledge a systematic preclinical SGN gene therapy study has yet to be performed. We hypothesized that more reliable SGN transduction could be achieved by administering AAV to the *scala tympani* that has a ~18x larger volume (1.81 µL in the gerbil) than Rosenthal's canal (0.10 µL) that is mainly occupied by SGNs 25.

**Figure 2:**
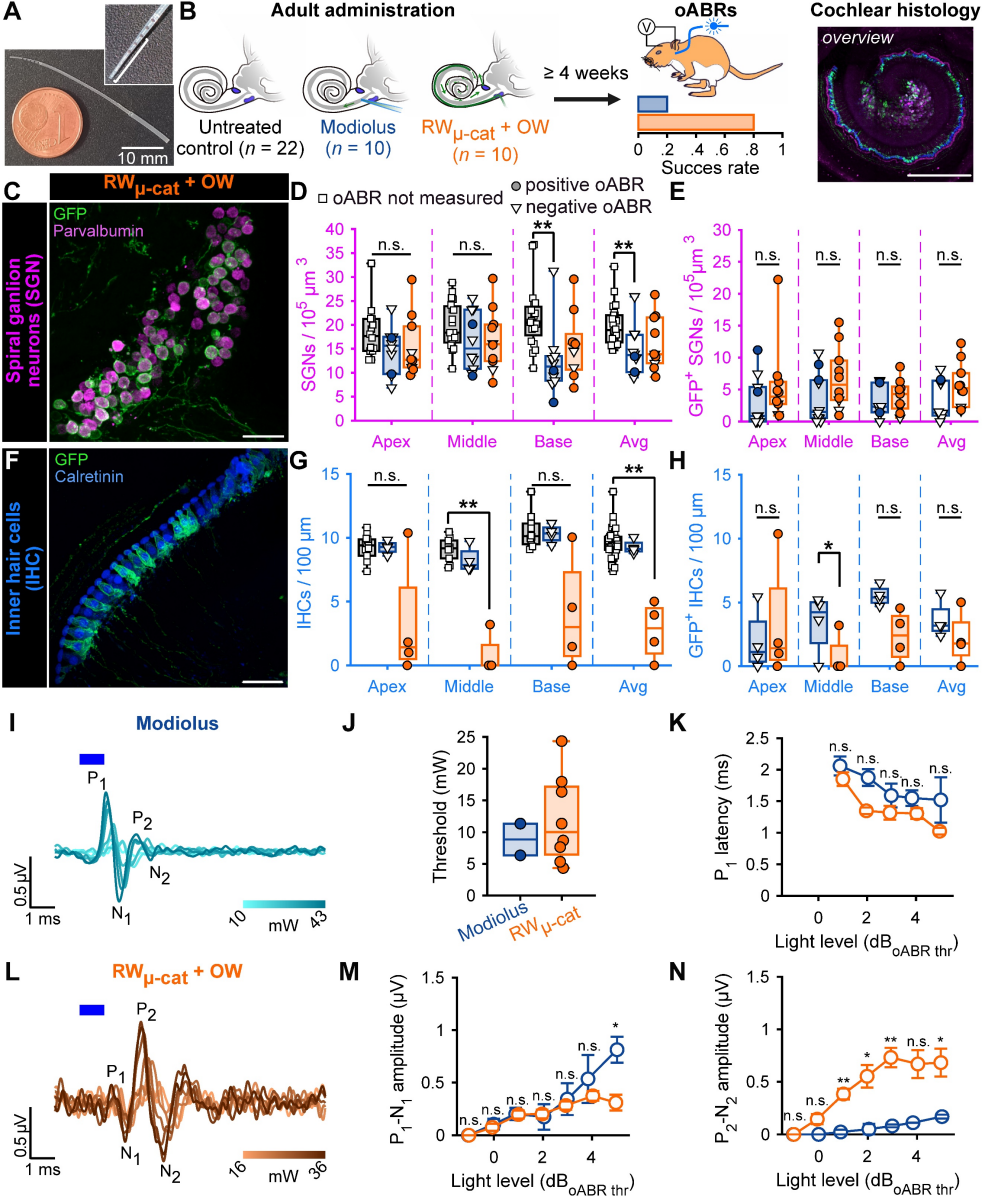
Viral administration with a micro-catheter inserted at the round window + vent at the oval window (RW_µ-cat_ + OW) mediates improved optogenetic modification of the SGNs compared to the reference modiolus injection. **A.** Picture of the catheter used for the RW_µ-cat_ + OW approach. The white line indicates the insertion depth (4.5-5 mm). **B.** Time course of the experiment. At adult age, animals were divided in three groups: i) untreated control (black); ii) modiolus injection (blue); iii) RW_µ-cat_ + OW administration (orange) of AAV-PHP.S-hSyn-CatCh. At least 4 weeks later, oABRs were measured. The proportion of animals for which a positive oABR were recorded are represented as boxplot. Next, the cochleae were collected for histology. Here, the overview image is a cross-modiolar section from a modiolus injected cochlea (scale bar = 500 µm). **C,F.** Representative maximum projection of confocal images obtained from immunolabelled cross-modiolar sections of spiral ganglion neurons (SGN, C, scale bar = 50 µm) and inner hair cells (IHC, F, scale bar = 50 µm). GFP (green) marks ChR-expressing cells. The SGNs were labelled with parvalbumin (magenta). The IHCs were labelled with calretinin (blue). **D-E, G-H.** Quantification of the SGN density (D, *n* = 6), GFP+ SGN density (E, *n* = 6), IHC density (G, *n* = 4) and GFP+ IHC density (H, *n* = 4). A filled circle was used when positive oABRs were measured and an open-triangle for the negative oABRs. No oABRs were measured from the untreated control cochleae (squared marker)..Kruskal-Wallis test followed by a multi-comparison test (n.s., non-significant; *, *P* ≤ 0.05; **, *P* ≤ 0.01). **I-L.** Representative oABRs recorded from modiolus injected (I) and RW_µ-cat_ + OW administered cochleae (L). The light intensity is color coded using the color scale in insert. **J.** Quantification of the oABR activation threshold measured from modiolus injected (orange, *n* = 2 positive oABR cochleae out of 10 injected ones) and RW_µ-cat_ + OW administred (blue, *n* = 8 positive oABR cochleae out of 10 injected ones) cochleae. **K,M-N.** Quantification of the P_1_ latency (K), P_1_-N_1_ amplitude (M) and P_2_-N_2_ amplitude (N) as a function of the light level relative to the oABR threshold. Box plots show minimum, 25^th^ percentile, median, 75^th^ percentile, and maximum. Averaged ± SEM. Wilcoxon rank sum test (n.s., non-significant; *, *P* ≤ 0.05; **, *P* ≤ 0.01). Approximately, 4 weeks after injection, animals were tested and expression of f-Chrimson-eYFP was analyzed by confocal microscopy of mid-modiolar cryosection immunolabeled for GFP and parvalbumin as a SGN marker, regardless of the presence or absence of optically evoked oABRs (**Figure S2B-C**). RW_µ-cat_ + vent administrations tended to enable higher SGN and GFP^+^ SGN densities (**Table 1**, **Figure S2B**) and transduction rates (**Table 1**, **Figure S2C**) compared to the reference modiolus injection. The RW_µ-cat_ + OW vent approach was selected for further investigation because, unlike the RW_µ-cat_ + PSCC vent approach, it limits active delivery of the viral suspension to the vestibule.

Here, we compared the modiolus injection with four distinct approaches employing micropump-driven AAV administration via microcatheter (µ-cat) provided by MED-EL (**Figure 2A**). The following approaches were tested: *i*) microcatheter (µ-cat) insertion via the round window (RW_µ-cat_); *ii*) RW_µ-cat_ and pressure relief by a vent at the oval window (RW_µ-cat_ + OW); *iii)* RW_µ-cat_ and pressure relief in the posterior semi-circular canal (RW_µ-cat_ + PSCC); *iv*) PSCC delivery and pressure relief evacuation vent drilled at the RW (PSCC + vent, **Figure S1**). In a preliminary set of experiments, those different approaches (RW_µ-cat_, *n* = 5; RW_µ-cat_ + PSCC, *n* = 7; RW_µ-cat_ + OW, *n* = 5; PSCC_cat_ + vent, *n* = 6) were compared to direct pressure modiolus injection (*n* = 6, **Figure S2A**) using AAV2/9-hSyn-f-Chrimson-eYFP (titer = 1-3 x 10^12^ GC/mL). In all those approaches, the viral suspension was mixed with fast-green (1:20) to visualize the evacuation of the suspension. Catheter-based AAV-administration was performed at 250 - 300 nL/min using a micropump and was stopped either after observing reflux of the suspension or after dosing 5 µL.

Approximately, 4 weeks after injection, animals were tested and expression of f-Chrimson-eYFP was analyzed by confocal microscopy of mid-modiolar cryosection immunolabeled for GFP and parvalbumin as a SGN marker, regardless of the presence or absence of optically evoked oABRs (**Figure S2B-C**). RW_µ-cat_ + vent administrations tended to enable higher SGN and GFP^+^ SGN densities (**Table 1**, **Figure S2B**) and transduction rates (**Table 1**, **Figure S2C**) compared to the reference modiolus injection. The RW_µ-cat_ + OW vent approach was selected for further investigation because, unlike the RW_µ-cat_ + PSCC vent approach, it limits active delivery of the viral suspension to the vestibule.

In order to further scrutinize the efficacy of the RW_µ-cat_ + OW delivery approach over the modiolus injection for optogenetic modification of the SGNs, we compared both approaches on a larger sample of animals (*n* = 10 per group) using AAV-PHP.S-hSyn-CatCh-eYFP (titer = 6.4 x 10^12^ GC/mL) against control non-injected cochleae (*n* = 22). Approximately, 4 weeks after administration, we tested for oABRs and expression of CatCh-eYFP was analysed by confocal microscopy of either mid-modiolar cryosection (*n* = 6 for both groups) or cross-modiolar section (*n* = 4 for both groups) immunolabeled for GFP, parvalbumin and calretinin (only for the cross-modiolar sections), regardless of the presence or absence of optically evoked oABRs (**Figure 2B**). The SGN density was reduced for both approaches compared to the control, **Figure 2C-D, Table 2**) and was significantly lower for the modiolus injected group (*P* = 0.0093, Kruskal-Wallis test followed by a Tukey-Kramer post-hoc test). The number of SGNs expressing CatCh-eYFP following RW_µ-cat_ + vent administration was 1.75-fold that of the modiolus injected cochleae (**Figure 2C,E, Table 2**). The RW_µ-cat_ + vent administration led to a significantly more homogenous SGN transduction than the modiolus injection (χ2 = 18.86, *P* = 1.41 x 10^-5^, Fligner-Killeen test for homogeneity of variances), thus supporting the superior reliability of the RW_µ-cat_ + vent approach to optogenetically modifying adult SGNs.

Here again, IHCs expressing CatCh-eYFP were found in both injection groups and thus IHC histology was performed from cochlear cross-modiolar slices. Quantification of the IHC density revealed a drastic loss of hair cells for the cochleae that underwent a RW_µ-cat_ + OW administration compared to the control cochleae (**Table 2**, *P* = 0.0027, Kruskal-Wallis test followed by a multi-comparison test).

oABRs were found in 20% of the animals subjected to intramodiolar injection and 80% of the animals undergoing RW_µ-cat_ + OW administration. oABRs activation thresholds were similar between both groups (8.85 ± 2.49 and 12.00 ± 2.46 mW for modiolus and RW_µ-cat_ + vent, respectively) but oABRs strongly differed in terms of wave morphologies. At 5 dB above the threshold, modiolus injected cochleae were characterized by a larger first oABR wave (P_1_-N_1_) than RW_µ-cat_ + vent administered cochleae (**Figure 2M**, 0.81 ± 0.12 and 0.31 ± 0.08 µV, *P* = 0.033, Wilcoxon rank sum test), but were also characterized by a ~1 order of magnitude smaller second wave (P_2_-N_2_, **Figure 2M**, 0.17 ± 0.02 and 0.68 ± 0.13 µV, *P* = 0.017, Wilcoxon rank sum test).

The increased reliability of SGN transduction - 80% of treated cochleae were oABR-positive and significantly more homogenous SGN transduction- together with a stronger activation of the auditory pathway - the larger 2^nd^ oABR wave reflecting activation of the auditory brainstem - make of the RW_µ-cat_ + OW approach a stronger candidate route of administration for optogenetic modification of the SGNs than the previously established intramodiolar AAV injection. Hence, in another set of experiments, we investigated whether the RW_µ-cat + OW_ approach could mediate SGNs transduction in a model of profound sensorineural hearing loss.

### Validation of RW_µ-cat_ + OW administration to mediate the transduction of the SGN in adult deafened cochleae

**Figure 3:**
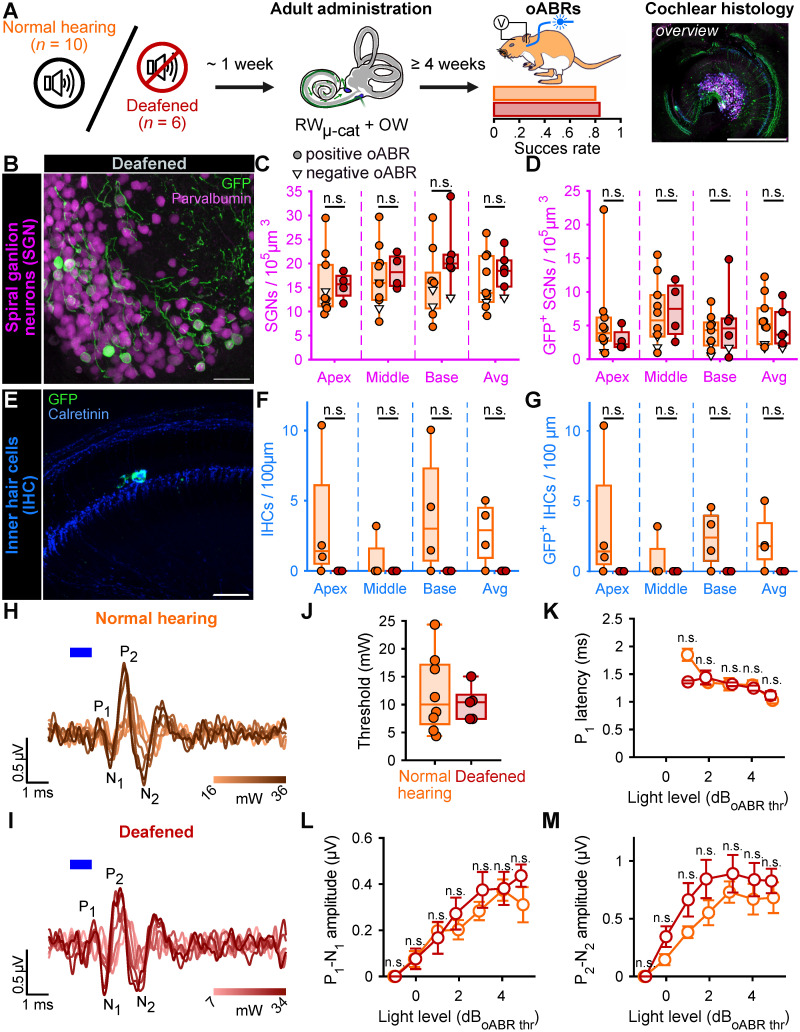
Viral administration with a RW_µ-cat_ + OW does not require the presence of inner hair cell to optogenetically modify the SGNs. **A.** Time course of the experiment. At adult age, a group of animals was deafened by cochlear round window injection of Kanamycin (100 mg/mL, *n* = 6). The control normal hearing animals are replotted from figure 2. One week after deafening, animals received a RW_µ-cat_ + OW administration of AAV-PHP.S-hSyn-CatCh. At least 4 weeks later, oABRs were measured. The proportion of animals for which a positive oABR were recorded are represented as boxplot. Next, the cochleae were collected for histology. Here, the overview image is a cross-modiolar section from a deafened cochlea (scale bar = 500 µm). **B,E.** Representative maximum projection of confocal images obtained from immunolabelled cross-modiolar sections of spiral ganglion neurons (SGN, B, scale bar = 50 µm) and inner hair cells (IHC, E, scale bar = 50 µm). GFP (green) marks ChR-expressing cells. The SGNs were labelled with parvalbumin (magenta). The IHCs were labelled with calretinin (blue). **C-D, F-G.** Quantification of the SGN density (C), GFP+ SGN density (D), IHC density (F) and GFP+ IHC density (G). A filled circle was used when positive oABRs were measured and an open-triangle for the negative oABRs. (n.s., non-significant) **H-I.** Representative oABRs recorded in normal hearing (H) and deafened (I) cochleae following RW_µ-cat_ + OW administration. The light intensity is color coded using the color scale in insert. **J.** Quantification of the oABR activation threshold measured from normal hearing (orange) and deafened (red) cochleae. **K-M.** Quantification of the P_1_ latency (K), P_1_-N_1_ amplitude (L) and P_2_-N_2_ amplitude (M) as a function of the light level relative to the oABR threshold. Box plots show minimum, 25^th^ percentile, median, 75^th^ percentile, and maximum (n.s., non-significant). Averaged ± SEM.

The main indication for cochlear implantation is to restore hearing in patients with profound sensorineural hearing loss characterized by absent or dysfunctional IHCs but remaining SGN somas 32. Therefore, we investigated whether the RW_µ-cat_ + vent administration could optogenetically modify the SGNs following pharmacological ablation of the IHCs and partial degeneration of the SGN dendrites. Induction of the profound hearing loss was achieved by injecting 3 µL of Kanamycin (100 mg/ml) into the cochlea by the RW one week before to RW_µ-cat_ + vent administration of AAV-PHP.S-hSyn-CatCh-eYFP (titer = 6.4 x 10^12^ GC/mL, *n* = 6 animals, **Figure 3A**).

Treated deafened cochleae were compared to the treated normal hearing cochleae described above (**Figure 2**). The success of the deafening procedure was confirmed by the absence of IHC observed at any turn of the kanamycin-injected cochleae by confocal imaging following immunolabeling (**Figure 3E-G**). The extent of the optogenetic modification was identical between normal hearing and deafened cochleae as shown by comparable GFP^+^ SGN densities (**Table 3**, **Figure 3B-D**) and oABRs (**Figure 3H-M**). Moreover, the deafening protocol did not reduce the total SGN density. Together those data confirm the utility of RW_µ-cat_ + OW vent viral vector delivery to optogenetically modify the SGNs in the naive and deafened cochlea.

Next, we investigated wheter the optogene therapy exhibited specificity towards SGN subtypes (**Figure S3**). To this end, the SGNs were co-stained with Calretinin, a marker of SGN type Ia/high-spontaneous rate neurons 33-36. In accordance with previous report 36, the proportion of Calretinin-positive SGNs was found to be the lowest at the base of the gerbil cochlea (**Figure S3A**). The ChR-expression rate was comparable between Calretinin-positive and -negative SGNs and amounted to 26.97 ± 1.84 and 30.57 ± 1.11%, respectively (**Figure S3B**, *P* = 0.68, Wilcoxon rank sum).

Finally, we examined a subset of 5 brains (3 normal hearing RW_µ-cat_ + OW and 2 deafened RW_µ-cat_ + OW) for the off-target expression of ChR (**Figure S4**). Histological analysis was performed on coronal slices at the level where axons from SGNs project to cochlear nucleus neurons (i.e., the first brain region exposed to the viral suspension after cochlear administration, 23). Expression of GFP was restricted to the cochlear nucleus on the injected side and was confined to the large axosomatic synapses on bushy cells (i.e., endbulbs of Held). Although this observation does not substitute for more extensive histologic analysis of larger brain regions, it does support the absence of major spread of AAV and off-target expression of ChR transduction upon the RW_µ-cat_ + OW administration of AAVs.

### Functional impact of CatCh expression in both IHCs and SGNs

In previous work with administration of AAV2/6, AAV-PHP.B and AAV-PHP.eB carrying ChRs under the control of hSyn promoter (titer between 2 x 10^12^ and 5 x 10^13^ GC/mL), the expression of ChR was limited to SGNs and no expression of ChR was reported in the IHCs 12-14,18,20. Here, using AAV-PHP.S with hSyn promoter at a similar titer (6.4 x 10^12^ GC/mL), we observed co-transduction of ChR in the IHCs in the majority of the treated adult cochleae. We therefore studied the opto-physiological relevance of IHC co-activation taking advantage of the comparison of cochlea with or without ChR-expressing IHCs.

**Figure 4:**
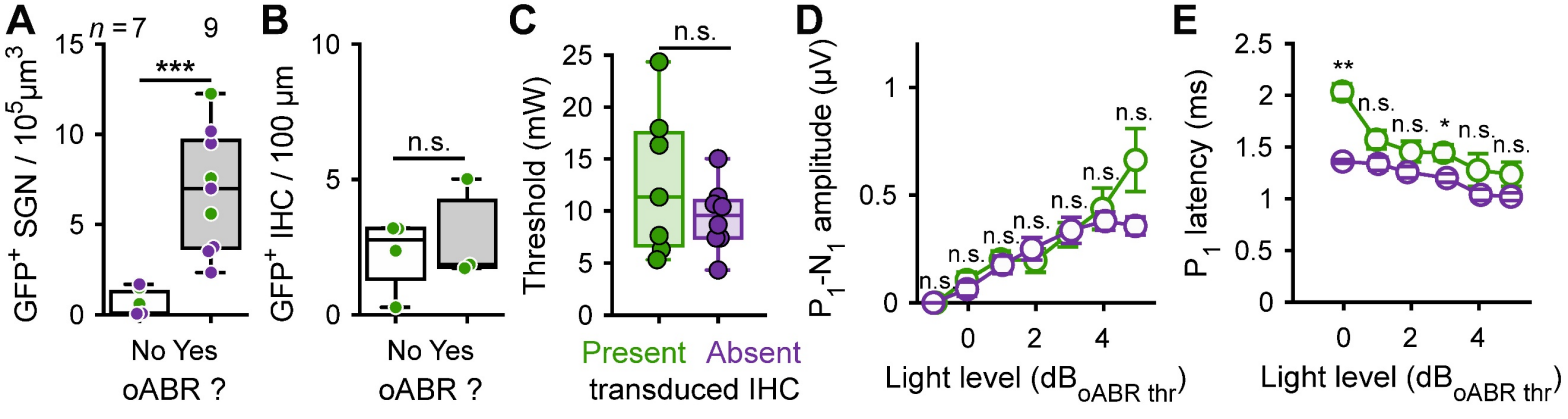
Contribution of optogenetically modified inner hair cell response to the oABRs. **A.** Quantification of the GFP^+^ SGN density as a function of the presence of the oABR response. **B.** For the cochleae where ChR-expressing IHC were observed, quantification of the IHC transduction as a function of the presence of the oABR. **C.** Quantification of the oABR threshold for cochlea where GFP+ IHC were present (green) or absent (purple). **D-E.** Quantification of the P_1_ latency (C), P_1_-N_1_ amplitude (D) as a function of the light level relative to the oABR threshold. Box plots show minimum, 25^th^ percentile, median, 75^th^ percentile, and maximum. Averaged ± SEM. Wilcoxon rank sum test (n.s., non-significant; *, *P* ≤ 0.05; **, *P* ≤ 0.01).

Firstly, we identified that at least 2.34 SGN/10^5^ µm^3^ (11.7% of the total amount of SGN in non-treated cochleae) were required to elicit an oABR (**Figure 4A**). The mean number of ChR-expressing SGNs was similar between positive oABR cochleae with or without ChR-expressing IHCs: 8.47 ± 1.97 SGN/10^5^ µm^3^ and 6.05 ± 1.37, respectively. The number of ChR-expressing IHC did not differ between oABR-positive and -negative cochleae (**Figure 4B**): 2.86 ± 1.86 and 2.26 ± 0.69 GFP^+^ IHC/100 µm, respectively. This suggests that oABRs are mainly caused by the photoactivation of the SGNs rather than IHCs.

Next, we investigated if the presence of ChR-expressing IHCs influenced the oABR properties. The oABR threshold radiant flux was similar for cochleae with and without ChR-expressing IHCs (**Figure 4C**): 12.76 ± 2.66 mW and 9.42 ± 1.13, respectively. At all light levels, the oABR amplitudes were similar between the cochleae with and without ChR-expressing IHCs (**Figure 4D**). Nonetheless, the oABR first wave latency tended to be longer for the cochlea with ChR-expressing IHCs and this effect was the strongest near threshold (**Figure 4E**, 1.36 ± 0.02 and 2.03 ± 0.08 ms, *P* = 0.009, Wilcoxon rank sum test).

## Discussion

Here we used mature Mongolian gerbils as a preclinical rodent model to further develop optogene therapy for the future optical cochlear implant. The Mongolian gerbil is a small rodent (~ 60-80 g) with a hearing frequency range similar to that of humans 37. Its low-frequency hearing is supported by a relatively large middle ear and cochlea, which allows cochlear implant and drug delivery studies to be conducted in better resemblance to the human ear than if conducted in high-frequency hearing rodents (e.g., mice and rats). We identified the AAV administration via a round window-inserted microcatheter with a pressure relief via an oval window vent to provide a reliable means for optogene therapy. Roughly 80% of the treated animals showed optogenetic activation of the auditory pathway compared to 40% with the previously described intramodiolar AAV injection (this study and 18). Throughout the study we probed the utility of the catheter-based viral vector administration for several combinations of AAV-capsids and ChRs, with a focus on AAV2/9 variants as well as on the ChRs CatCh and f-Chrimson with previously documented good membrane targeting. We identified substantial off-target expression in IHCs but no obvious ChR expression in the brainstem. Optogenetic stimulation of the auditory pathway required ≥10% of SGNs to express ChR and was robust to the absence of ChR expressing IHCs (e.g., lack of transduction or kanamycin deafening). Together this study advances optogenetic hearing restoration by demonstrating the utility of a clinically established AAV administration route and studying the impact that hair cell stimulation could have on optogenetic hearing restoration in cases with residual low frequency hearing.

### Old and new standards for evaluating SGN gene therapies

The standard routine to assess the activation of the SGNs *in vivo* consists of measuring the auditory brainstem response (ABR). Acoustic and optical ABRs are characterized by 1 to 3 waves with the first wave reflecting the synchronous activation of the SGNs 28,38. Previous studies have shown that oABRs are a good proxy for ChR transduction of SGNs and faithfully report the optogenetic activation of the auditory pathway 14,18. In fact, recordings from single SGNs, single cochlear nucleus neurons, inferior colliculus and auditory cortex demonstrate propagated activity elicited by optogenetic stimulation of SGNs 8,18,21.

In previous studies developing optogenetic modification of SGNs, IHCs transduction was not observed in mid-modiolar cryosections of the cochlea, where only a few IHCs are seen per turn 14,18. Using AAV.PHP-S and *scala tympani* delivery, our initial observations suggested that transduction of IHCs was systematic (further discussed below). Therefore, we developed the semi-thick (220 µm) cross-modiolar sections to visualize the SGNs and the IHCs they innervate from the same section. This approach appeared to be a good intermediate step between confocal imaging of few thin cryosections (~20 µm, e.g. 14,18) and comprehensive analysis by light sheet microscopy of cleared intact cochleae 25. Indeed, the thick sections allow to preserve the anatomical integrity of the cochlea while providing a good antibody and light penetration for staining and imaging, respectively, two aspects that are critical for light sheet fluorescence microscopy.

Another critical aspect in the development of cell and gene therapy in the cochlea is the automated and reproducible evaluation of the effect of the therapy. In addition, preclinical development requires the evaluation of hundreds to thousands of samples. Therefore, we developed a pipeline in Arivis 4D using Cell Pose and a custom SGN model 27,39 to segment and quantify SGN survival and transduction rate. This allowed a large number of samples to be processed using batch analysis with minimal time and human supervision.

### Design of the viral vector construct

First-in-human clinical data for cochlear gene therapy were recently reported for Otoferlin-related auditory synaptopathy patients who regained hearing by replacing the missing *OTOF* gene 2,3,31. These proof-of-concept studies are helping pave the way for the optogenetic hearing restoration.

Achieving efficient and safe ChR expression in adult SGNs is far from trivial and requires identification of the optimal combination of viral capsid promoter, ChR, regulatory sequences, viral titer, and approach to viral vector delivery in the cochlea. Previous and current studies in the cochlea rely on *in vivo* screening of different viral constructs 13,14,19,20. Lessons learned from previous works include: *i*) human synapsin (hSyn) promoter mediates efficient 12,18,43 and safe 17 ChR expression in the SGNs; *ii*) f-Chrimson 12,20 is a potential ChR candidate for hearing restoration; *iii*) f-Chrimson membrane expression can be restored after removing of GFP if replaced with trafficking sequences 19; *iv*) f-Chrimson membrane expression is reduced when transduction occurs at adult age 14. In this study, hSyn was used in conjunction with ChR fused to eYFP, for histological detection of CatCh in the tissue. The blue-light-activated ChR2 variant CatCh has proven utility for restoring auditory activity and percepts in deafened mammals 18. Moreover, CatCh is well suited for blue LED-array stimulation of the auditory nerve achieving near-physiological frequency selectivity 9,10. The red-light-activated Chrimson variant f-Chrimson shows fast-closing kinetics enabling a higher temporal fidelity of optogenetic SGN stimulation than CatCh 12,20.

To take into account the potential passage of viral vector suspension to the brain following cochlear administration 23, that could lead to transduction of central nervous system neurons, the capsid AAV.PHP-S was used for its narrow tropism towards the peripheral nervous system 24 and cochlear cells 44. The viral vector suspension was administered at the titer at which it was produced: 6.4 x 10^12^ gene copies/mL, consistent with previous reports using adult gerbils 8,14,18.

### Validating AAV-PHP.S to deliver ChR to the SGNs

The ability of AAV-PHP.S-hSyn-CatCh to drive ChR expression in the SGNs was first evaluated by intracochlear pressure injection in early postnatal age gerbils. AAV-PHP.S successfully drive ChR expression in the SGNs of both cochleae. Bilateral transduction following early postnatal injection is normal 12-15,20,21 and is discussed to occur through viral vector spread via the cochlear aqueduct and/or the endolymphatic duct to the cerebrospinal fluid space 45. Surprisingly, the SGN density was twofold decreased in the injected cochleae compared to the non-injected ones, suggesting a potential AAV dose- or protein level-dependent toxicity leading to SGN loss 17,19. The precise folding and trafficking of proteins across membranes is complex but crucial, especially in the context of microbial opsin expression 46. Impairment of these processes could potentially lead to toxic levels of cell stress through aggregation in the endoplasmic reticulum, trafficking defects and/or aggregate formation. Thus, cell-damaging stress may result from strong overexpression of the optogenetic actuator. Another surprise was to find IHC expressing GFP^+^ in the injected cochlea, even though IHCs do not natively express synapsin 47,48. Transcription of the protein in absence of a promoter naturally at work in IHCs is another suggestion of the highly potent transduction occurring with AAV-PHP.S-hSyn-CatCh (6.4 x 10^12^ gene copies/mL). Optimization of the dose at which AAV-PHP.S-hSyn-CatCh should be injected, at a neonatal stage, to maximize transduction of SGNs was beyond the scope of this study. Despite transduction of, both, SGNs and IHCs, we found loss of SGNs but not IHCs. We do not currently comprehend the reasons for the different consequences, which might include cell-type specific toxicity of virus, transgene expression or immune response.

### Improved viral vector-delivery to the adult SGNs using microcatheter round window administration and vent

Achieving reliable ChR expression in adult SGNs is key to clinical translation of the oCI. Previous work in adult gerbils found that pressure injection into the modiolus of AAV2/6-hSyn-CatCh enabled optogenetic modification of ~30% of SGNs in approximately 40% of treated cochleae 18. Following work with other capsids and/or ChRs injected directly into the modiolus of gerbil cochleae, lower performance was reported in terms of transduction rate (~10%) and proportion of successfully treated cochleae (~10%, 14,22). Alternatively, attempts to inject the viral suspension directly into the perilymph either through the round window or via the posterior semicircular canal have not proven to be a reliable alternative 15.

Is the direct injection of the AAV-PHP-S-hSyn-CatCh into the modiolus sufficient to improve the success of the treatment? Using it, oABRs could be measured in 20% of the treated cochleae for an overall transduction rate of 25.69% of all injected cochleae. Because of methodological differences, comparisons are complicated but it is reasonable to assume that AAV.PHP-S-hSyn-CatCh performs at least similarly than in previous reports 18,22. Nevertheless, this approach remains an interesting route, perhaps for cases where AAV re-dosing is required. It is worth noting that direct modiolus injection has been shown to be feasible in human temporal bone 30, where clear and visible landmarks are available.

As an alternative, we investigated if microcatheter-based pump-controlled administration approaches via the *scala tympani* could offer a viable alternative to the direct modiolus injection. Here, we demonstrated a fourfold increase in the success rate of measuring oABRs (80 vs 20%), as well as an 8% increase in SGN survival (~81 vs 73%), a 1.75-fold increase in transduction rate (28 vs 16%) and a significant increase of reliability of the RW_µ-cat_ + vent administration compared to modiolus injection to optogenetically modify adult SGNs. This improvement comes at the cost of a drastic loss of IHC, most likely due to the insertion and withdrawal of the catheter used to deliver the viral suspension 49. Additionally, we cannot exclude that AAV-PHP.S transduction or ChR expression might have exerted IHC toxicity. The field of clinical cochlear implants has developed several robotic and automated approaches for minimally invasive insertion 50. These approaches could be adapted for human catheter-based viral delivery if IHC survival is required. An oval window vent via stapedotomy is a procedure used in several clinical trials on cochlear gene therapy 2,3,31 and is unlikely to have majorly contributed to IHC loss. In a large fraction of treated cochleae with AAV.PHP-S-hSYn-CatCh, the remaining IHCs were expressing ChR. For both modiolus injection and microcatheter administration, the IHC transduction rate amounted to ~38 and 30% for modiolus and RW_µ-cat_ + vent administration, respectively. The fact that no oABRs were measured from the modiolus-injected cochleae, where SGN transduction was close to 0%, supports that IHC optogenetic activation alone was not sufficient to drive synchronous firing in the SGNs. Moreover, the likelihood of viral particles reaching the SGNs via the IHCs seemed low, considering that pharmacologic depletion of the IHCs by intracochlear injection of kanamycin one week prior to viral vector delivery did not affect the performance of the procedure. Co-transduction of IHCs and SGNs was found in RW_µ-cat_ + vent treated cochleae, and their optical co-activation was associated with a slightly longer latency of the SGN compound action potential. As previously suggested, our data support that the contribution of optogenetically activated hair cells, if any, is very small compared to that of SGNs 22.

Why is *scala tympani* administration more efficient for transducing SGNs than direct injection into the modiolus where their cell bodies are located? One answer may be found in the available volume of the scala tympani: it is about 18 times larger than the modiolus 25, which is already densely packed with SGN cell bodies. Although the route by which the virus finds its way to the SGN can only be speculated at this point, our data suggest that an increased number of virus particles find their way to the SGN with RW_µ-cat_ + vent administration compared to direct modiolus injection. In the present study, a maximum volume of 5 µL was administered, corresponding to ~80% of the cumulated volume of the scala timpani, vestibuli and media 25. This might suggest for mammals with bigger cochlea to scale up the volume of administered viral suspension to the volume of those cumulated cavities that would translate to ~15 µL and ~71 µL for guinea pig and humans, respectively.

### Lessons learned for optogenetic hearing restoration and limitations of the study

This study indicates that AAV administration via the round window with pressure relief at the round window is a reliable approach to transduce SGNs. A similar approach has recently been successfully employed in clinical trials on AAV-mediated gene therapy of otoferlin-related auditory synaptopathy 2,3,31. There, IHCs have been the target that can be considered more accessible to virus administered to the *scala tympani* than SGNs that, except for their peripheral neurites innervating the organ of Corti, are thoroughly encased in the bony modiolus. Here we employed microcatheters that are smaller versions of catheters approved for clinical use and delivered volumes that relatively speaking come close to those applied to the human cochlea. Clearly, the reliability and efficacy of the optogene therapy need further optimization to provide optogenetic modification of the spiral ganglion in 100% of treated cochleae and in the majority of the SGNs. We note that 30% seems a good target of ChR^+^ positive cells given the experience of the more advanced vision restoration 51. In fact, this expression rate has also supported demonstration of improved coding of spectral information in prior preclinical work on the waveguide or LED-based oCI stimulation of SGNs 8,9. However, eventually, the benefits of oCI are predicted to be greatest with the largest possible population of ChR^+^ SGNs, indicating the need for further efforts to enhance the efficacy of optogene therapy e.g. by capsid library screens and directed evolution 42,52. This seems of paramount importance for transfer of the method to non-human primates and future clinical translation to be achieved. In fact, a recent study employed a round-window catheter approach to deliver AAV.PHP.eB to express the ChRmine variant ChReef under the control of the synapsin promotor in SGNs of non-human primates (marmosets 53). Proof of concept of optogenetic activation of the auditory pathway was obtained, yet only in one out of nine animals indicating insufficient efficacy of the viral gene transfer. Moreover, future longitudinal studies of catheter based optogene therapy of mature animals should evaluate the stability of ChR expression in analogy to previous work on early postnatal gene therapy in mice 17. Finally, our deafness model has limitations in that it primarily represents acute deafness and might not resemble aspects of chronic deafness such as organ of Corti scarring and partial or complete loss of SGNs.

Off-target expression of the therapeutic construct, opsins in the case of optogenetic therapy, can cause adverse or confounding effects. We observed that despite choosing viral vector and promotor for specifically targeting SGNs, the only neural population with somatas contained in the cochlea, expression of ChR also occurred in hair cells to different extent. While this calls for further efforts for more selective optogenetic modification, it also provided us an opportunity to compare optogenetic stimulation of the auditory pathway with and without ChR expressing IHCs. This is relevant for future optogenetic hearing restoration in cases of residual low-frequency hearing currently currently treated with electroacoustic or hybrid stimulation of the eCI 54,55. In this particular case, a shorter microcatheter should be considered to deliver the viral suspension to the cochlea while sparing the apical hair cells. Our data suggest minimal impact of potential optogenetic stimulation of remaining IHCs on optically induced activity of the auditory pathway. We did not find differences in thresholds or amplitudes of the oABRs, but found a slightly delayed latency of the first oABR wave, which is consistent with optogenetically induced glutamate release from IHCs 56 contributing to SGN firing. The potential contribution of light-evoked glutamate release from IHCs should be further investigated by electrocochleography or single SGN recordings, potentially in the presence and absence of blockers of glutamatergic synaptic transmission 18,57. If remaining hair cells were transduced and optogenetically co-stimulated SGNs, this might affect temporal coding and would need to be considered by sound coding strategies. The scope of our current study did not cover a comprehensive study of ChR expression across the brain or analysis of viscera. We note that different from a previous study employing early postnatal AAV injection into the mouse cochlea that showed ChR expression in various brain regions, the neural ChR expression upon microcatheter based AAV administration to the mature cochlea of the present study was largely limited to SGNs. This is promising and encourages comprehensive toxicology studies in preparation of clinical trials.

## Materials and methods

### Animals

Optogenetic data were obtained from 61 and control histological data from 22 Mongolian gerbils of either sex. For all procedures, animals were kept on a retro-controlled heating pad and their body temperature maintained at 37 degrees Celsius. All experiments were performed following the guidelines provided by the German national animal care and were approved by the board for animal welfare of the University Medical Center Göttingen and the animal welfare office of the state of Lower Saxony (LAVES). Animals were kept in a 12 hours light/dark cycle, with access to food and water *ad libitum*.

### Viral vector production

Viral vector employed in this study were produced as previously described 58. Briefly, pHelper plasmid (TaKaRa, USA), the trans-plasmid with PHP.S capsid or AAV2/9 capsid and the *cis*-plasmid with CatCh under the control of the human synapsin promotor were triple transfected with HEK-293T cells. Cells were regularly checked for mycoplasma contamination. Viral particles were collected 72 hours post-transfection from the medium and 120 hours post-transfection from both the cells and the medium. They were then treated by precipitation using 40% polyethylene glycol 8000 (Acros Organics, Germany) in 500 mM NaCl for 2 hours at 4°C. Following centrifugation, these particles were merged with cell pellets for subsequent processing. Cell pellets were suspended in 500 mM NaCl, 40 mM Tris, 2.5 mM MgCl2, pH 8, and 100 U/ml of salt-activated nuclease (Arcticzymes, USA) at 37 °C for 30 min. Subsequently, the cell lysates underwent clarification through centrifugation at 2000×g for 10 minutes and purification through iodixanol (Optiprep, Axis Shield, Norway) step gradients (15%, 25%, 40%, and 60%) at 350,000×g for 2.25 hours. The viruses were concentrated using Amicon filters (EMD, UFC910024) and then suspended in sterile phosphate-buffered saline (PBS) supplemented with 0.001% Pluronic F-68 (Gibco, Germany). Using an AAV titration kit (TaKaRa/Clontech), the viral vector titers were measured, by determining the number of DNase I resistant vg using qPCR (StepOne, Applied Biosystems). Silver stainings (Pierce, Germany) were used to check for the purity of the viruses routinely after gel electrophoresis (Novex™ 4-12% Tris-Glycine, Thermo Fisher Scientific). The presence of viral capsid proteins was then confirmed in all viral vector preparations. The viral stocks were stored at -80 °C until needed.

### Early postnatal injections in the *scala tympani*

Injections were performed as previously described 14,58. Briefly, under general Isoflurane anaesthesia (1.5 - 5%) and analgesia (subcutaneous injection of buprenorphine, 0.1 mg/kg and carprofen, 5 mg/kg), the left bulla of Mongolian gerbils of P7-8 was exposed via a retro-auricular incision. A volume of 1 - 1.5 µL of viral vector suspension, mixed with fast green (1:20), was loaded into a quartz micropipette (tip diameter approximately 20 µm, Science products; pulled with a P-2000 laser puller, Sutter Instruments) connected to a pressure microinjector (100-125 PSI, PLI-100 pico injector, Harvard Apparatus) and injected in the *scala tympani*. The right micropipette placement was confirmed visually by a perilymph reflux in the pipette tip when entering the *scala tympani*. After injection, the pipette was carefully retracted, the tissue above the injection site was repositioned and the wound was sutured. Animals were then daily tracked for the first 3 days and then weekly observed until oABRs were recorded. Carprofen (5 mg/kg) was given the first 3 days, and additional application could be performed at later point.

### Adult modiolus injections

Injections were performed as first described by Wrobel and colleagues 18. Briefly, under general Isoflurane anaesthesia (1.5 - 5%) and analgesia (subcutaneous injection of buprenorphine, 0.1 mg/kg and carprofen, 5 mg/kg), the left bulla of Mongolian gerbils of at least 8 weeks was exposed via a retro-auricular incision. A bullostomy was performed to expose the round window niche and a hole was drilled in the modiolus. A volume of 3 µL of viral vector suspension was subsequentially injected in the drilled hole using a quartz micropipette (same design as described in early postnatal injection). After the injection, the tissue above the bulla was repositioned and the wound was sutured. Animals were then daily tracked for the first 3 days and received additional subdermal carprofen (5 mg/kg) injections. Later, animals were weekly tracked.

### Adult *scala tympani* administrations

Administrations were performed similarly than the modiolus injection previously described at the difference that no hole was drilled in the modiolus. In this case, a microcatheter provided by the cochlear implant manufacturer MED-EL was filled with 5 µL of viral vector suspension filled with fast green (1:20) and connected to a micro-infusion pump (UltraMicroPump3, Word Precision Instrument, United States of America, **Figure S1A-B**). If required by the procedure, an evacuation vent was drilled next to the oval window or in the posterior semi-circular canal. After insertion of the microcatheter in the RW (4.5 - 5.5 µm deep, **Figure S1B-C**), the viral vector was administered at 250 - 300 nL/min and the administration was stopped either following reflux of the viral suspension or when the 5 µL were dispensed. For the procedure to be successful, it was necessary to deeply insert the microcatheter into the *scala tympani*. Typically, the administration took 20 mins. Following careful retraction of the microcatheter, the procedure was finished as described for the modiolus injections.

### Deafening

The deafening procedure was achieved under general Isoflurane anaesthesia (1.5 - 5%) and analgesia (subcutaneous injection of buprenorphine, 0.1 mg/kg and carprofen, 5 mg/kg). The left bulla of Mongolian gerbils of at least 8 weeks was exposed via a retro-auricular incision and a bullostomy was performed to expose the round window niche. A volume of 3 µL of kanamycin (100 mg/ml, CarlRoth Gmbh, T832.2) was subsequentially injected through the round window membrane using a quartz micropipette (same design as described in early postnatal and adult modiolus injection). After the injection, the tissue above the bulla was repositioned and the wound was sutured. Animals were then daily tracked for the first 3 days and received additional subdermal carprofen (5 mg/kg) injections. From the second week after injection, animals were weekly tracked and additional application of carprofen (5 mg/kg) could be performed.

### Stimulation

Stimuli were generated via a custom-made system based on NI-DAQ-Cards (NI PCI-6229, National Instrument, Austin, USA) and custom-written MATLAB scripts (The MathWorks, Natick, USA). Acoustic stimuli were generated at a sampling rate of 830 kHz and presented via an open-field speaker (Avisoft Inc., Germany) placed at ~15 cm from the left pinna. A ¼ inch microphone and amplifier (D4039; 2610; Brüel & Kjaer GmbH, Naerum, Denmark) were used to calibrate sounds. Optical stimuli were generated at a sampling rate of 50 kHz and were delivered into the cochlea via an optical fiber (ø = 200 µm, 0.39 NA, Thorlabs GmbH, Germany) coupled to a blue laser (473 nm, MLLFN-473-100, 100 mW, Changchun New Industry Optoelectronics). The fibre insertion angle has been chosen to maximize optical stimulation of all cochlear turns 8. The maximum radiant flux at the output of the optical fiber was calibrated before every experiment (LaserCheck; Coherent Inc.) and later used for calibration. Access to the round window was gained similarly than for the viral vector injection described above. Stimulations were presented at 20 Hz at least 500 times per tested condition for auditory brainstem stimulation and 100 times for compound action potentials.

### Auditory brainstem recordings

At least 8 weeks after early postnatal injection and 4 weeks after adult injection/administration, auditory brainstem responses (ABR) were recorded. The ABRs were recorded using a custom-made differential amplifier with sub-dermal needle electrodes placed at the vertex, below the ipsilateral pinna and at the contralateral leg. Potentials were digitalized using the same NI-DAQ-Cards than described above for stimulation at a sampling rate of 50 kHz. Response to every stimulation presentation were saved for offline analysis. ABR analysis was performed using custom-made Matlab scripts: traces were filtered (2^nd^ order Butterworth pass-band filter between 300 and 3000 Hz) and averaged per tested condition. Wave detection (wave I, II and III) was achieved manually and the activation threshold defined as the condition for which the smallest ABR could be visually detected. Amplitude of the different waves was obtained by detecting automatically the negative peak following each wave.

### Compound action potential recordings

For a subset of animals, compound action potentials (CAP) were recorded using a custom-made differential amplifier with a silver ball electrode place at the round window niche and two sub-dermal needle electrodes placed below the ipsilateral pinna and at the contralateral leg. Potentials were digitalized using the same NI-DAQ-Cards than described above for stimulation at a sampling rate of 50 kHz. Response to every stimulation presentation were saved for offline analysis. ABR analysis was performed using custom-made Matlab scripts: traces were filtered (2^nd^ order Butterworth pass-band filter between 100 and 10000 Hz) and averaged per tested condition. The first negative was automatically detected if its amplitude was lower than -1 µV and the activation threshold defined as the condition for which the smallest CAP was detected.

### Cochlea and brain harvesting

At the end of the CAP/ABR recordings, animals were sacrificed by cervical dislocation under deep anaesthesia and their cochlea and brain were immediately collected and fixed in formaldehyde 3.7% (Carl Roth, Germany). Tissue fixation was done for 1 hour and at least for 24 hours for cochleae and brains, respectively. Cochleae were then decalcified in 0.12 M EDTA for 7 days.

### Mid-modiolar cryosection of the cochlea

Cochleae were first dehydrated by incubating them overnight in 30% sucrose solution in PBS. Next, slices were embedded in Cryomatrix (Epredia, United Stated of America) and 25 µm slices were obtained using a cryostat (Leica CM3050S, Germany) using a cutting angle parallel to the modiolus. After staining, slices were mounted with Mowiol 4-88 (Carl Roth, Germany) and imaged using a LSM 510 Zeiss confocal microscope (Zeiss, Germany) with a 40x objective. A z-stack image (1 µm z-resolution) was taken per cochlear turn.

### Cross-modiolar section of the cochlea

Cochleae were sliced using a vibratome (Leica 1200S speed = 0.02 mm/s, amplitude = 0.6 mm) with a thickness of 220 µm at a cutting angle perpendicular to the modiolus. For staining, one slice per cochlear turn was kept on the basis of visualizing the IHCs and the SGNs innervating them. After staining, slices were cleared and mounted in FOCM 59. A 30 µm z-stack (SGNs: 1 µm z-resolution; IHCs: 3 µm z-resolution) from the SGNs and IHCs were taken using a Leica SP8 confocal microscope (Leica microsystems, Germany) with a 40x objective. Overview images were taken using the same microscope equipped with a 20x objective and using the inbuilt tile function.

### Cochlear histology

Permeabilization and blocking of the slices was obtained by 2 hours incubation in 16% goat serum dilution buffer (16% normal goat serum, 450 mM NaCl, 0.6% Triton X-100 20 mM phosphate buffer, pH 7.4). Information about the primary antibody incubation is summarized in **Table 4**. Staining with secondary antibodies was done in one hour for mid-modiolar cryosections and 48 hours for cross-modiolar sections at 4°C using goat anti-chicken 488 (A11039, Life Technologies, 1:200), goat anti-guinea-pig 568 (A11075, Life Technologies, 1:200) and goat anti-rabbit 647 (A21244, Life Technologies, 1:200).

### Histological analysis

Analysis of mid-modiolar images was done using a custom-made script in Matlab as previously described in 14,21. The conversion from SGN density in 2D to 3D was done by multiplying the surface in which SGN were segmented by the number of stacks that were imaged and the z-resolution.

Analysis of cross-modiolar images was done within Vision 4D (Zeiss, Germany). SGN somas were automatically segmented using a custom model in Cellpose 2.0 27,39. The volumetric boundary containing all segmented SGNs was then computed using a custom-written function. Identification of the GFP positive SGNs was done as previously described in 14,21, using a python script directly in Vision 4D. IHCs and ChR-expressing IHCs were counted manually in Fiji from the maximum projection along a manually drawn line. ChR-expressing IHCs were visually identified as cells brighter than the mean + 2 standard deviation of the GFP background signal measured from a non-neural region of the image.

### Data analysis and statistics

All data were analyzed using MATLAB (MathWorks). Averages were expressed in figures and main text as mean ± SEM. For statistical comparison between two independent groups, data were tested for normality (Jarque-Bera test) and Student's t-test or Wilcoxon rank sum test were used accordingly. For statistical comparison between more than two independent groups, data were tested for normality (Jarque-Bera test), and one-way ANOVA or a Kruskal-Wallis test was applied accordingly following by a Tukey-Kramer post-hoc test.

## Supplementary Material

Supplementary figures.

## Figures and Tables

**Table 1:** Cochlear histological quantification of modiolus injected, RW_µ-cat_, RW_µ-cat_ + PSCC, RW_µ-cat_ + OW and PSCC_cat_ + vent administred cochleae.

	Modiolus(*n* = 6)	RW_µ-cat_(*n* = 5)	RW_µ-cat_ + PSCC (*n* = 7)	RW_µ-cat_ + OW (*n* = 5)	PSCC_cat_ + vent (*n* = 6)
**SGN density (/10^5^ µm^3^)**	11.61 ± 0.78	16.85 ± 1.82	16.59 ± 0.97	13.69 ± 1.52	19.17 ± 1.46
**GFP+ SGN density (/10^5^ µm^3^)**	1.04 ± 0.79	0.72 ± 0.49	2.11 ± 0.65	1.52 ± 0.34	1.14 ± 0.5
**SGN absolute transduction rate (%)**	8.05 ± 5.66	2.86 ± 0.89	12.79 ± 3.21	11.90 ± 2.60	1.53 ± 1.02

**Table 2:** Cochlear histological quantification from untreated control, modiolus and RW_µ-cat_ + OW AAV-treated cochleae. The survival rate refers to the ratio of SGN density between a given group and the controls. The absolute transduction is the ratio between the number of GFP+ cells and number of cells within a group. The relative transduction rate is the ratio between the number of GFP+ cells from a given group and the average number of cells measured from untreated control cochleae.

	Control (C, *n* = 22)	Modiolus (M, *n* = 10)	RW_µ-cat_ + OW (R, *n* = 10)	Statistics
**SGN density (/10^5^ µm^3^)**	20.00 ± 0.91	14.55 ± 1.60	16.13 ± 1.77	**C** vs **M**: *P* = 0.009**C** vs **R**: *P* = 0.08
**SGN survival rate (%)**		72.72 ± 8.02	80.63 ± 8.83	*P* = 0.57
**GFP+ SGN density (/10^5^ µm^3^)**		3.24 ± 0.98	5.64 ± 1.11	*P* = 0.12
**SGN absolute transduction rate (%)**		25.69 ± 7.59	33.71 ± 4.89	*P* = 0.31
**SGN relative transduction rate (%)**		16.21 ± 4.90	28.21 ± 5.56	*P* = 0.12
**IHC density (/100 µm)**	9.58 ± 0.21	9.27 ± 0.27	2.71 ± 1.12	**C** vs **M**: *P* = 0.79**C** vs **R**: *P* = 0.0027
**IHC survival rate (%)**		96.78 ± 2.41	28.25 ± 10.08	*P* = 0.0286
**GFP+ IHC density (/100 µm)**		3.62 ± 1.46	2.15 ± 2.09	*P* = 0.2
**IHC absolute transduction rate (%)**		39.36 ± 7.10	81.19 ± 15.36	*P* = 0.11
**IHC relative transduction rate (%)**		37.79 ± 6.61	29.90 ± 9.17	*P* = 0.2

**Table 3:** Cochlear histological quantification from normal hearing and deafened cochleae following RW_µ-cat_ + OW treatment.

	Normal hearing - RW_µ-cat_ + vent	Deafened - RW_µ-cat_ + vent	Statistics
**SGN density (/10^5^ µm^3^)**	16.13 ± 1.77	18.33 ± 1.64	*P* = 0.43
**SGN survival rate (%)**	80.63 ± 8.83	91.64 ± 8.21	*P* = 0.43
**GFP+ SGN density (/10^5^ µm^3^)**	5.64 ± 1.11	4.63 ± 1.22	*P* = 0.56
**SGN absolute transduction rate (%)**	33.71 ± 4.89	24.00 ± 4.78	*P* = 0.22
**SGN relative transduction rate (%)**	28.21 ± 5.56	23.16 ± 6.13	*P* = 0.56
**IHC density (/100 µm)**	2.71 ± 1.12	0 ± 0	*P* = 0.067
**IHC survival rate (%)**	28.25 ± 11.65	0 ± 0	*P* = 0.067
**GFP+ IHC density (/100 µm)**	2.15 ± 2.09	0 ± 0	*P* = 0.067
**IHC absolute transduction rate (%)**	81.19 ± 16.29		
**IHC relative transduction rate (%)**	22.43 ± 10.91	0 ± 0	*P* = 0.067

**Table 4:** Summary of primary antibody incubation per slicing approach.

Marker of	Primary antibody reference	Mid-modiolar cryosection	Cross-modiolar section
GFP	chicken anti-GFP #ab13970, Abcam, USA	1:500, overnight at 4°C	1:500, 72 hours at 4°C
SGNs	guinea pig anti-parvalbumin 195004, Synaptic Systems, Germany	1:300, overnight at 4°C	1:200, 72 hours at 4°C
IHCs	rabbit anti-calretinin 7697, Swant, Switzerland	NA	1:500, 72 hours at 4°C
