# Supplementary material for: Improved optogenetic modification of spiral ganglion neurons for future optical cochlear implants
Source: Theranostics. 2025 Mar 18;15(10):4270–86. doi: 10.7150/thno.104474 (PMC11984395; doi:10.7150/thno.104474)
Supplement: Supplementary file 1 — Supplementary figures. [file thnov15p4270s1.pdf]

Improved optogenetic modification of the spiral ganglion neurons for future optical cochlear implants

Anupriya Thirumalai[1,2,3,4], Jana Henseler[1,2,3], Marzieh Enayati[1,2,3], Kathrin Kusch[1,2,5,6,7], Roland Hessler[8], Tobias Moser[1,2,5,7,9,10§], Antoine Tarquin Huet[1,2,3,5,8,9,11§]

[1]Institute for Auditory Neuroscience, University Medical Center Göttingen, 37075 Göttingen, Germany;

[2]InnerEarLab, University Medical Center Göttingen, 37075 Göttingen, Germany

[3]Auditory Circuit Lab, University Medical Center Göttingen, 37075 Göttingen, Germany

[4]Göttingen Graduate School for Neurosciences and Molecular Biosciences, University of Göttingen, Göttingen, Germany

[5]Auditory Neuroscience and Optogenetics Laboratory, German Primate Center, Göttingen, Germany

[6]Functional Auditory Genomics group, University Medical Center Göttingen, 37075 Göttingen, Germany

[7]Else Kroener Fresenius Center for Optogenetic Therapies, University Medical Center Göttingen, 37075 Göttingen, Germany

[8]MED-EL GmbH, Innsbruck, Austria

[9]Cluster of Excellence "Multiscale Bioimaging: from Molecular Machines to Networks of Excitable Cells" (MBExC), University of Göttingen, Göttingen, Germany

[10]Auditory Neuroscience and Synaptic Nanophysiology Group, Max Planck Institute for Multidisciplinary Sciences, Göttingen, Germany

[11]Current address: Institute for Neurosciences Montpellier, Institut National de la Santé et de la Recherche Médical, University of Montpellier, Montpellier, France

[§]Those authors contributed equally.

Corresponding authors: tmoser@gwdg.de, antoine.huet@inserm.fr

Supplementary information

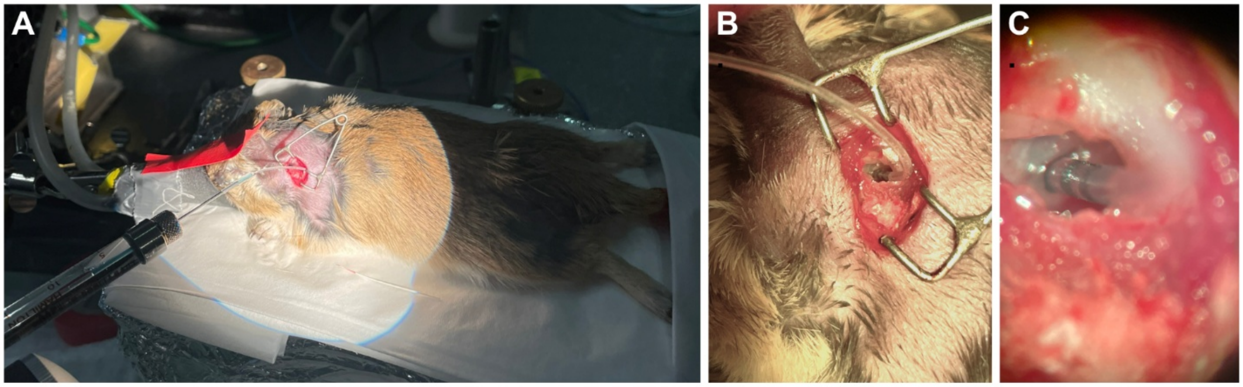

Figure S1. Procedure to perform catheter viral suspension delivery into the gerbil cochlea.

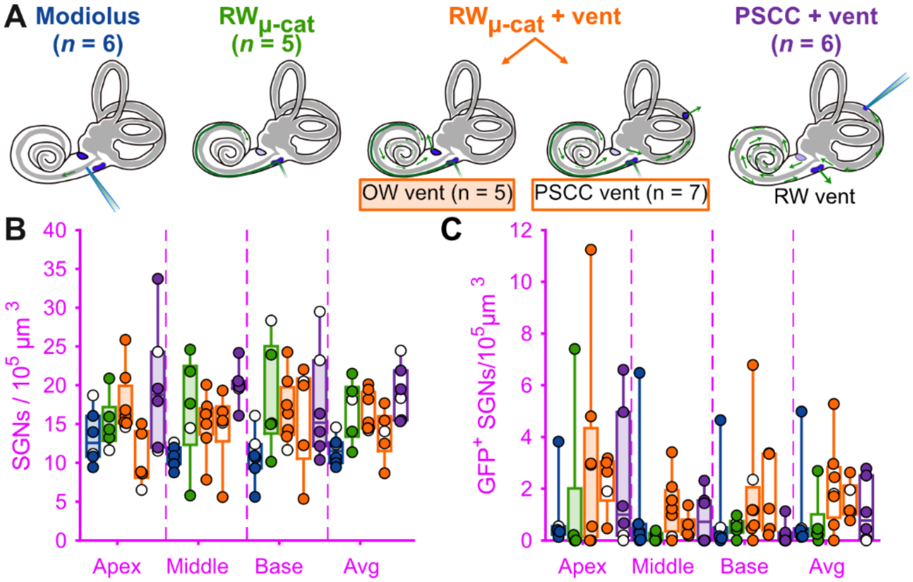

Figure S2. Comparison of viral administration approaches in the adult gerbil cochlea. **A.** Schematic representation of the different administration approaches (see Materials and methods for details). **B-C.** Quantification of the SGN density (B) and GFP$^+$ SGN density (C) from the injected cochleae with the different administration approaches presented in A. Filled markers were used when positive oABRs were measured and an open-marker for the negative oABRs. Box plots show minimum, 25th percentile, median, 75th percentile, and maximum. Averaged ± SEM. Kruskal-Wallis test followed by Tukey-Kramer post-hoc test (*, $P \leq 0.05$; **, $P \leq 0.01$).

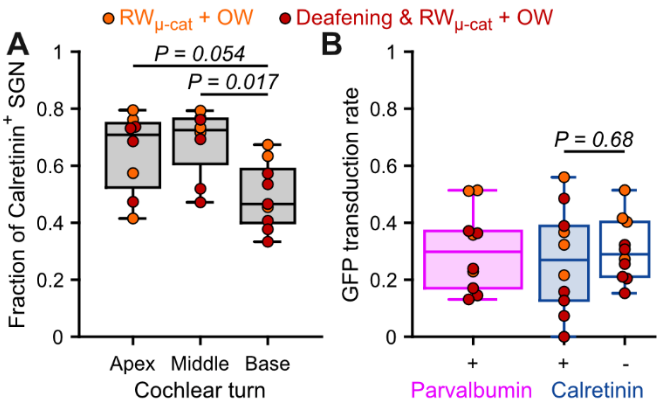

Figure S3. Subtypes unspecific optogenetic modification of the SGNs using AAV-PHP.S-hSyn-CatCh-eYFP. **A.** Quantification of the fraction of Calretinin-positive SGNs ('type Ia SGNs'), i.e., the ratio of the number of Calretinin- and Parvalbumin-positive cells, at the 3 cochlear turns following $RW_{\mu\text{-cat}}$ + OW administration. Kruskal-Wallis test followed by a multi-comparison test. The markers filled in orange correspond to normal-hearing and in red to deafened cochleae. **B.** Quantification of the ChR-expression rate from Parvalbumin-positive SGNs (magenta), and from Calretinin-positive (+, blue filling) and -negative (-, blue, white filling). Wilcoxon rank sum test. Box plots show minimum, 25th percentile, median, 75th percentile, and maximum.

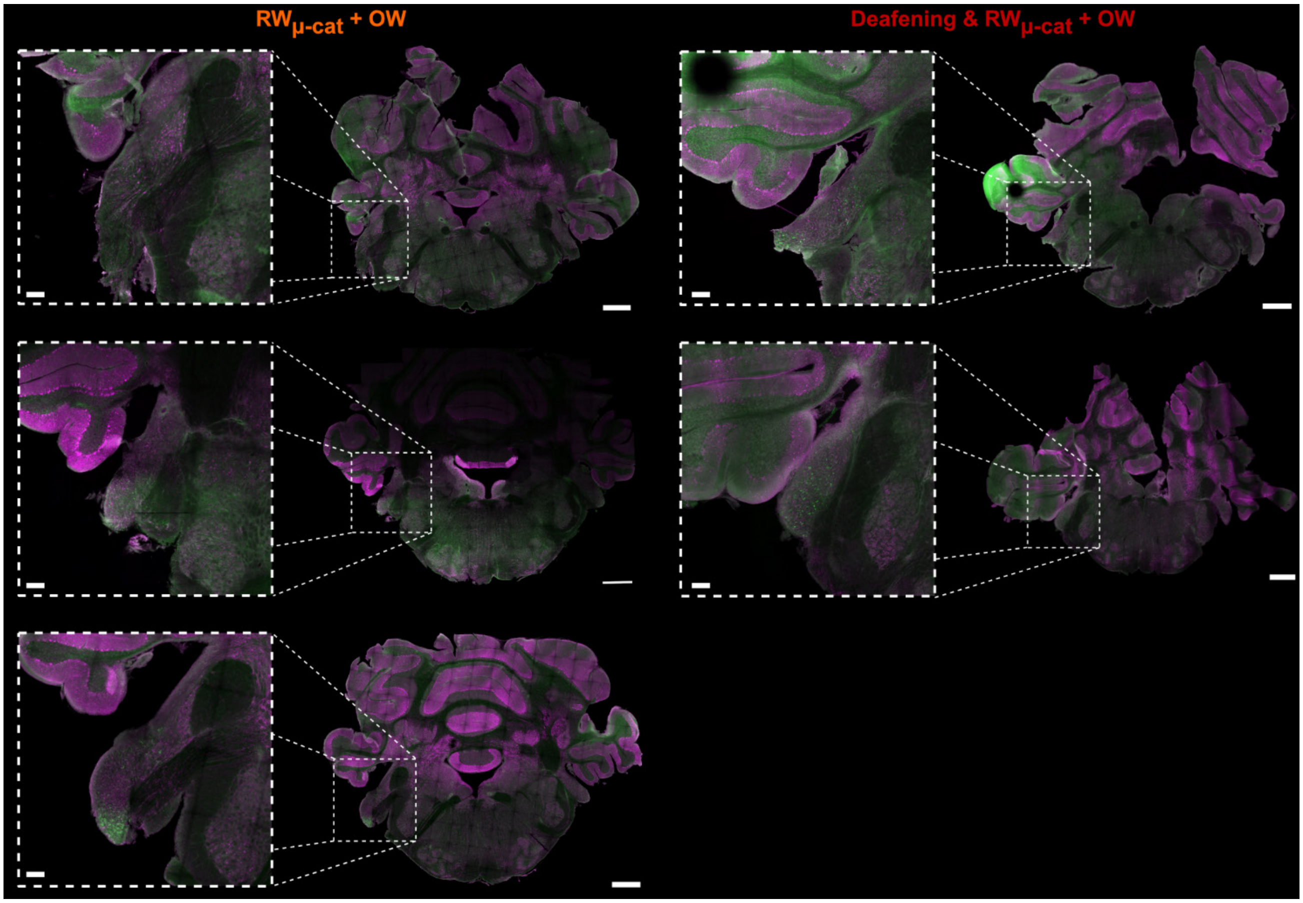

Figure S4. Absence of transduction in the central nervous system following $RW_{\mu\text{-cat}}$ + vent AAV-administration. Coronal slices of 5 gerbil brains following $RW_{\mu\text{-cat}}$ + vent AAV-administration. Slices were stained for parvalbumin (purple) and GFP (green), scale bar = 1 mm. The insert shows a magnification of the antero-ventral cochlear nucleus where GFP signal is found in axons of the SGNs but not in the neurons on which they project (scale bar = 0.2 mm).
